# Distinct Aqueous Humour Cytokine Profiles of Patients with Pachychoroid Neovasculopathy and Neovascular Age-related Macular Degeneration

**DOI:** 10.1038/s41598-018-28484-w

**Published:** 2018-07-12

**Authors:** Nobuhiro Terao, Hideki Koizumi, Kentaro Kojima, Tetsuya Yamagishi, Yuji Yamamoto, Kengo Yoshii, Koji Kitazawa, Asako Hiraga, Munetoyo Toda, Shigeru Kinoshita, Chie Sotozono, Junji Hamuro

**Affiliations:** 1 Department of Ophthalmology, Kyoto Prefectural University of Medicine, Kyoto, Japan; 2 Department of Ophthalmology, University of the Ryukyus, Okinawa, Japan; 3 Department of Mathematics and Statistics in Medical Sciences, Kyoto Prefectural University of Medicine, Kyoto, Japan; 4 Department of Frontier Medical Science and Technology for Ophthalmology, Kyoto Prefectural University of Medicine, Kyoto, Japan

## Abstract

This study investigated the pathophysiological features of pachychoroid neovasculopathy (PNV) and neovascular age-related macular degeneration (nAMD) by analysing and comparing cytokine profiles in aqueous humour (AH) collected from 18 PNV, 18 nAMD and 11 control patients. Responses to intravitreal injection of aflibercept were also analysed in the PNV and nAMD groups. In the PNV group, vascular endothelial growth factor (VEGF)-A was significantly lower than in the nAMD group (*p* = 0.03) but was almost identical to that in the control group (*p* = 0.86). The nAMD group showed positive correlations between interleukin (IL)-6 and IL-8 (*r* = 0.78, *p* < 0.001), IL-6 and monocyte chemoattractant protein (MCP)-1 (*r* = 0.68, *p* = 0.002) and IL-8 and MCP-1 (*r* = 0.68, *p* = 0.002). In the nAMD group, eyes with dry maculae one month after the first aflibercept injection showed significantly lower VEGF-A and placental growth factor (PlGF) at baseline than those with wet maculae (*p* = 0.02 for both). However, there was no significant difference between dry and wet maculae in the PNV group. The results suggest that angiogenic factors and proinflammatory cytokines may play the distinct roles in the pathogenesis of PNV and nAMD.

## Introduction

Pachychoroid neovasculopathy (PNV) is a newly proposed clinical entity with type-1 (subretinal pigment epithelium [sub-RPE]) choroidal neovascularisation (CNV), characterised by choroidal abnormalities (i.e., pachychoroid) accompanied by thickened choroid, large dilation of choroidal vessels and choroidal vascular hyperpermeability (CVH)^1^. The epidemiology, diagnostic criteria and pathophysiology of PNV are currently unknown; therefore, it is clinically treated using the same protocols used for neovascular age-related macular degeneration (nAMD) as a peculiar subtype.

Patients with a history of central serous chorioretinopathy (CSC) develop type-1 CNV^2,3^, including polypoidal choroidal vasculopathy (PCV)^4^. In 2012, Fung *et al*.^5^ reported 22 cases with type-1 CNV occurred in eyes with long-term CSC.; in 2013, the same group proposed a clinical definition of pachychoroid pigment epitheliopathy (PPE), which has CSC-like characteristics but does not involve a history of serous retinal detachment^6^. In 2015, Fung *et al*. proposed that type-1 CNV with CSC and PPE characteristics be called pachychoroid neovasculopathy^1^ because all cases described in these reports showed common cardinal features, including the presence of choroidal thickening, dilation of choroidal vessels, CVH and lack of drusen.

Miyake *et al*.^7^ examined cases of nAMD using new diagnostic criteria for PNV and reported that 19.5% of nAMD cases met this new definition of PNV. They found that the age of PNV onset was younger than that of nAMD, and allele frequencies of ARMS2A69 and CFH162V polymorphisms, which are disease susceptibility genes of nAMD, were also significantly different between PNV and nAMD. This suggests that there are epidemiological and genetic differences between the two disease spectra.

The disease spectrum associated with pachychoroid includes PPE, CSC, PNV and PCV^1^. Pang *et al*.^1^ claimed that PCV is not a distinct entity in itself but rather a manifestation of longstanding type-1 neovascularisation, with the causative disease being irrelevant. There have been several reports describing PCV with CVH, which may account for distinct responses to treatment^4,8^, and these reports imply that because CVH and choroidal thickening are important disease features, treatment response and prognosis of PNV may differ from those of nAMD. Thus, differences in the epidemiologies, disease susceptibility genes and treatment responses suggest that PNV and nAMD may have different pathophysiologies.

It is widely accepted that the activation of proinflammatory cytokines and subsequent upregulation of angiogenic factors, especially vascular endothelial growth factor (VEGF), play an important role in the development of CNV in nAMD. Previously, many reports have described the analysis of cytokines in eyes with nAMD^9^; in studies using aqueous humour (AH), various inflammatory cytokines, such as interferon-γ-induced protein (IP)-10, interleukin (IL)-8, monocyte chemoattractant protein (MCP)-1 and C-reactive protein (CRP), and angiogenic factors, such as angiogenin, VEGF and angiopoietin 2, have been shown to be related to CNV in nAMD^10–12^. In contrast, the pathophysiology of PNV is elusive, and the cytokine profile in AH has not been elucidated. There is only one report mentioning the association of VEGF with PNV^13^.

In this study, the cytokine profiles in AH of PNV and nAMD were comprehensively analysed to identify differences in cytokine profiles between these diseases. In addition, associations between observed cytokine profiles and responses to intravitreal injection of aflibercept were analysed in PNV and nAMD groups.

## Results

AH was collected from 18 PNV and 18 nAMD patients with type-1 CNV and 11 control patients. Table 1 shows the demographic and clinical characteristics of the subjects included in this study. There were no significant differences in age, sex or axial length among the three groups, and no significant differences were observed in the best-corrected visual acuity (BCVA), completion of posterior vitreous detachment (PVD), the presence of a polypoidal lesions, the greatest linear dimension (GLD) and CNV lesion size between the PNV and nAMD groups. The median subfoveal choroidal thickness was significantly thicker in the PNV group (median: 332 μm, interquartile range: 279–392 μm) than in the nAMD group (242 μm, 167–365 μm; *p* = 0.03).

**Table 1:** Demographic and Clinical Characteristics among the Three Groups.

	Pachychoroid neovasculopathy	Neovascular AMD	Control	
	*n* = 18	n = 18	n = 11	*p*-value
Age, y	68.5 (59.0–74.8)	73.5 (69.0–76.3)	75.0 (64.0–80.0)	0.16*
Female sex, n (%)	4 (22.2%)	5 (27.8%)	3 (27.3%)	1.00^†^
Axial length, mm	23.71 (22.96–24.79)	23.11 (22.63–24.07)	23.85 (23.48–24.95)	0.06*
BCVA, logMAR	0.30 (0.10–0.40)	0.35 (0.15–0.52)	NA	0.43^‡^
Subfoveal choroidal thickness, μm	332 (279–392)	242 (167–365)	NA	**0.03** ^‡^
Complete PVD, n (%)	5 (27.8%)	10 (55.6%)	NA	0.18^†^
Polypoidal lesion, n (%)	4 (22.2%)	9 (50.0%)	NA	0.16^†^
Greatest linear dimension, µm	3080 (2225–4619)	2717 (1740–3375)	NA	0.41^‡^
CNV lesion size, mm^2^	4.14 (1.22–7.32)	1.74 (0.74–4.17)	NA	0.24^‡^

Values are the median (interquartile range).

*P* < 0.05 was considered significant (bold).

*Kruskal-Wallis test.

^†^Fisher’s exact test.

^‡^Wilcoxon signed-rank test.

NA = not available.

AMD = age-related macular degeneration; BCVA = best-corrected visual acuity; CNV = choroidal neovascularisation; logMAR = logarithm of the minimal angle of resolution; PVD = posterior vitreous detachment.

**Figure 1:**
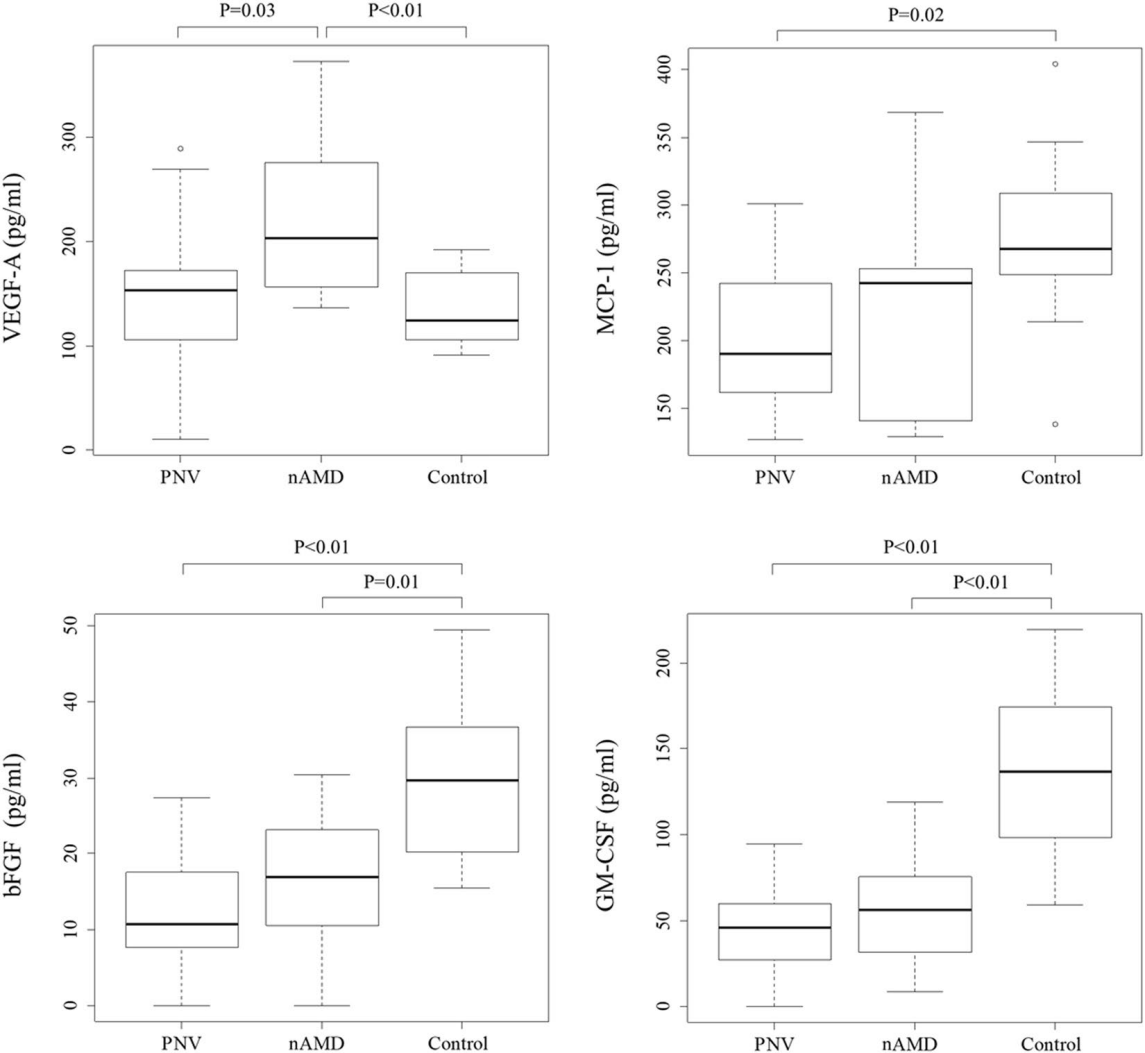
Baseline median concentrations of vascular endothelial growth factor (VEGF)-A, monocyte chemoattractant protein (MCP)-1, basic fibroblast growth factor (bFGF) and granulocyte macrophage colony-stimulating factor (GM-CSF) among the three groups. The concentrations of VEGF-A were significantly higher in the neovascular age-related macular degeneration (nAMD) group compared to the pachychoroid neovasculopathy (PNV) and control groups (*p* = 0.02 and *p* = 0.01, respectively). The boxes represent the median and interquartile ranges, with the line in the middle of the boxes corresponding to the median value.

Among the 30 cytokines analysed in this study, 10 cytokines (i.e., basic fibroblast growth factor [bFGF], granulocyte macrophage colony-stimulating factor [GM-CSF], IL-1 receptor antagonist [IL-1ra], IL-6, IL-8, IP-10, MCP-1, macrophage inflammatory protein [MIP]-1β, VEGF-A and PlGF) were detected in all three groups, while the remaining 20 cytokines were below detectable levels. The detected cytokine levels are summarised in Table 2. There were significant differences in MCP-1 (*p* = 0.01), bFGF (*p* < 0.01), GM-CSF (*p* < 0.01) and VEGF-A (*p* < 0.01) concentrations among the three groups, which were determined using the Kruskal-Wallis test. Figure 1 shows the results of the Steel-Dwass analysis of these four cytokines among the three groups. VEGF-A in the PNV group was significantly lower than in the nAMD group (*p* = 0.03), but it was almost identical to that in the control group (*p* = 0.86). MCP-1 was significantly higher in the control group compared to the PNV group (*p* = 0.02), but it was almost identical to that in the nAMD group. BFGF was significantly higher in the control group compared to both the PNV and nAMD groups (*p* < 0.01 and *p* = 0.01, respectively), while GM-CSF was significantly higher in the control group compared to both the PNV and nAMD groups (*p* < 0.01 and *p* < 0.01, respectively).

**Table 2:** Levels of Ten Cytokines in the AH among the Three Groups.

	Pachychoroid neovasculopathy	Neovascular AMD	Control	
	*n* = 18	*n* = 18	*n* = 11	*p*-value*
IL-1ra, pg/ml	97.13 (31.71–204.12)	93.52 (11.69–238.09)	231.11 (120.64–295.89)	0.16
IL-6, pg/ml	2.96 (2.10–10.80)	6.90 (3.62–12.31)	3.40 (2.46–3.89)	0.10
IL-8, pg/ml	5.53 (3.25–6.81)	6.44 (4.84–9.78)	6.80 (4.32–8.33)	0.19
MCP-1, pg/ml	190.01 (155.43–247.69)	242.24 (139.99–255.59)	267.56 (245.73–321.87)	**0.01**
MIP-1β, pg/ml	15.93 (11.65–20.20)	15.92 (14.46–25.64)	15.13 (9.16–27.15)	0.72
IP-10, pg/ml	152.98 (74.51–231.74)	183.60 (147.90–280.66)	146.15 (87.31–195.22)	0.09
bFGF, pg/ml	10.75 (6.87–18.28)	16.95 (10.23–23.47)	29.61 (17.88–37.51)	**<0.01**
GM-CSF, pg/ml	46.19 (27.05–61.31)	56.61 (30.69–76.18)	136.87 (96.37–183.51)	**<0.01**
VEGF-A, pg/ml	153.78 (103.76–178.14)	203.21 (154.88–277.66)	124.53 (101.44–173.02)	**<0.01**
PlGF, pg/ml	4.30 (2.67–5.81)	4.63 (3.61–6.77)	4.08 (2.29–5.62)	0.36

Values are the median (interquartile range).

*P* < 0.05 was considered significant (bold).

*Kruskal-Wallis test.

AMD = age-related macular degeneration; IL-1ra = interleukin 1 receptor antagonist; IL = interleukin; MCP-1 = monocyte chemoattractant protein 1; MIP-1β = macrophage inflammatory protein 1β; IP-10 = interferon-γ-induced protein 10; bFGF = basic fibroblast growth factor; GM-CSF = granulocyte macrophage colony-stimulating factor; VEGF-A = vascular endothelial growth factor A; PlGF = placental growth factor. Other cytokines were below the detection limit.

**Figure 2:**
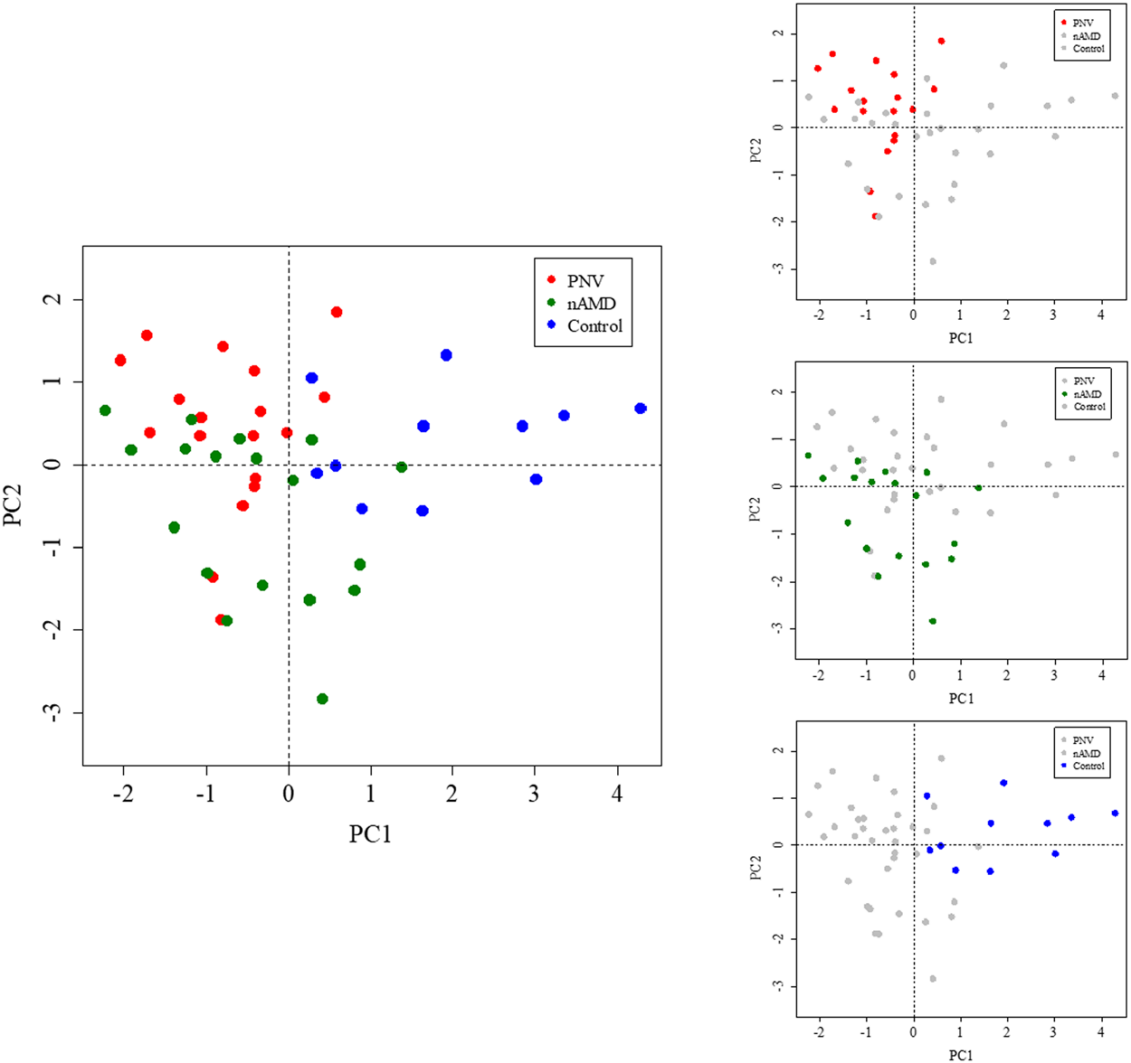
Principal component (PC) analysis of the data from four cytokines (VEGF-A, MCP-1, bFGF and GM-CSF). Neovascular age-related macular degeneration (nAMD; green), pachychoroid neovasculopathy (PNV; red) and control (blue) are plotted in a space defined by PCs 1 and 2, which are the two best axes representing the entire dataset. In the PC-1 axis, the control group is well separated from the other two groups. PNV and nAMD are distinguished by the PC-2 axis. The results of PC analysis indicate that the three pathological conditions form clusters and can be distinguished by four types of cytokines. VEGF-A = vascular endothelial growth factor A; MCP-1 = monocyte chemoattractant protein 1; bFGF = basic fibroblast growth factor; GM-CSF = granulocyte macrophage colony-stimulating factor.

The Spearman’s rank correlation coefficients among the 10 cytokines in the PNV and nAMD groups are summarised in Table 3. In the PNV group, there were positive correlations between IL-8 and MIP-1β (*r* = 0.65, *p* = 0.004), IP-10 and VEGF-A (r = 0.59, *p* = 0.01), bFGF and GM-CSF (r = 0.58, *p* = 0.01), VEGF-A and PlGF (r = 0.58, *p* = 0.01) and IL-1ra and IP-10 (r = 0.54, *p* = 0.02). There were negative correlations between IL-1ra and GM-CSF (r = −0.58, *p* = 0.01), IL-1ra and bFGF (r = −0.53, *p* = 0.02) and GM-CSF and PlGF (r = −0.50, *p* = 0.04). In the nAMD group, there were positive correlations between bFGF and GM-CSF (r = 0.90, *p* < 0.001), IL-6 and IL-8 (*r* = 0.78, *p* < 0.001), IL-6 and MCP-1 (*r* = 0.68, *p* = 0.002), IL-8 and MCP-1 (*r* = 0.68, *p* = 0.002), MCP-1 and MIP-1β (r = 0.53, *p* = 0.02) and IL-6 and MIP-1β (r = 0.49, *p* = 0.04). There was a negative correlation between MIP-1β and GM-CSF (r = −0.49, p = 0.04). Figure 2 shows the results of the principal component (PC) analysis of the data from the four cytokines (i.e., VEGF-A, MCP-1, bFGF and GM-CSF), which differed significantly among the three groups based on the Kruskal-Wallis test. Cytokine mapping showed distinguishable profiles among the PNV, nAMD and control groups, while a PC analysis identified the first two PCs, which explained 77.1% of the variation in the dataset; the control group was separated from the PNV and nAMD groups by PC 1. In addition, the PNV and nAMD groups were separated by PC 2, and each group formed its own cluster.

**Table 3:** Spearman’s Rank Correlation Coefficient for Ten Cytokines in Patients with Pachychoroid Neovasculopathy (a) and Neovascular Age-related Macular Degeneration (b).

	IL-1ra	IL-6	IL-8	MCP-1	MIP-1β	IP-10	bFGF	GM-CSF	VEGF-A
**Pachychoroid neovasculopathy**									
IL-6	−0.03	1							
IL-8	0.11	0.34	1						
MCP-1	0.38	0.4	0.38	1					
MIP-1β	−0.07	0.23	**0.65** ^†^	0.39	1				
IP-10	0.54*	0.18	0.2	0.35	0.22	1			
bFGF	−0.53*	0.23	0.41	−0.09	0.35	−0.2	1		
GM-CSF	−0.58*	−0.04	0.03	−0.1	0.15	−0.44	0.58*	1	
VEGF-A	0.19	0.38	0.35	0.13	0.25	0.59*	0.26	−0.22	1
PlGF	0.26	0.37	0.43	0.01	0.12	0.44	0.23	−0.50*	0.58*
**Neovascular age-related macular degeneration**									
IL-6	−0.09	1							
IL-8	−0.08	**0.78** ^‡^	1						
MCP-1	−0.14	**0.68** ^†^	**0.68** ^†^	1					
MIP-1β	−0.21	0.49*	0.32	0.53*	1				
IP-10	−0.31	0.2	0.12	0.18	0.42	1			
bFGF	0.4	0.04	0.14	0.1	−0.41	0.12	1		
GM-CSF	0.38	−0.15	0.01	0.05	−0.49*	−0.03	**0.90** ^‡^	1	
VEGF-A	<0.01	0.14	0.25	−0.1	−0.28	−0.01	0.46	0.32	1
PlGF	0.19	0.16	0.21	−0.19	−0.2	−0.34	0.14	0.13	0.17

*P < 0.05, ^†^*p* < 0.01, ^‡^*p* < 0.001, *p* < 0.05 were considered significant (bold).

IL-1ra = interleukin 1 receptor antagonist; IL = interleukin; MCP-1 = monocyte chemoattractant protein 1; MIP-1β = macrophage inflammatory protein 1β; IP-10 = interferon-γ-induced protein 10; bFGF = basic fibroblast growth factor; GM-CSF = granulocyte macrophage colony-stimulating factor; VEGF-A = vascular endothelial growth factor A; PlGF = placental growth factor. Other cytokines were below the detection limit.

Table 4 summarises the factors associated with dry maculae after the first intravitreal aflibercept injection. One month after the initial treatment, dry maculae was achieved in 10 of 18 eyes (55.6%) in the PNV group, whereas 9 of 18 eyes (50.0%) attained dry maculae in the nAMD group. In the PNV and nAMD groups, there were no significant differences in the associated factors between dry and wet maculae. Table 5 shows the concentrations of the 10 cytokines in PNV and nAMD, categorised according to the maculae status one month after the first aflibercept intravitreal injection. In the nAMD group, there were significant differences in the concentrations of VEGF-A (dry maculae: 166.88, 147.07–201.60 pg/ml vs. wet maculae: 275.98, 207.66–298.00 pg/ml, *p* = 0.02) and PlGF (3.67, 3.09–4.63 pg/ml vs. 5.99, 4.58–7.30 pg/ml, *p* = 0.02) between dry and wet maculae. In contrast, the PNV group showed no significant differences in cytokines between dry and wet maculae.

**Table 4:** Factors Associated with Dry Macula after the First Aflibercept Injection in Patients with Pachychoroid Neovasculopathy and Neovascular Age-related Macular Degeneration.

	Pachychoroid neovasculopathy			Neovascular age-related macular degeneration		
	Dry (n = 10)	Wet (n = 8)	*p*-value	Dry (n = 9)	Wet (n = 9)	*p*-value
Age, y	71.0 (59.0–80.75)	64.0 (56.5–70.75)	0.17*	73.0 (68.0–76.0)	74.0 (69.5–76.5)	0.60*
Female sex, n (%)	2 (20.0%)	2 (25.0%)	1.00^†^	2 (22.2%)	3 (33.3%)	1.00^†^
Axial length, mm	23.71 (22.96–24.79)	23.61 (22.94–24.91)	0.92*	23.01 (22.65–24.63)	23.17 (22.23–243.82)	0.50*
BCVA, logMAR	0.40 (0.14–0.47)	0.13 (0.10–0.37)	0.18*	0.30 (0.19–0.52)	0.40 (0.07–0.55)	0.53*
Subfoveal choroidal thickness, μm	343 (309–417)	305 (240–357)	0.21*	242 (193–370)	228 (163–338)	0.57*
Complete PVD, n (%)	3 (30.0%)	2 (25.0%)	1.00^†^	5 (55.6%)	5 (55.6%)	1.00^†^
Polypoidal lesion, n (%)	4 (40.0%)	0 (0%)	0.09^†^	5 (55.6%)	4 (44.4%)	1.00^†^
Greatest linear dimension, µm	3277 (2808–4444)	2107 (1422–5091)	0.25*	2336 (1638–3290)	3155 (1671–4110)	0.57*
CNV lesion size, mm^2^	4.41 (2.05–7.32)	3.42 (0.56–8.23)	0.66*	1.92 (0.76–3.63)	1.47 (0.71–5.76)	0.97*

Values are the median (interquartile range).

*Wilcoxon signed-rank test.

^†^Fisher’s exact test.

BCVA = best-corrected visual acuity; CNV = choroidal neovascularisation; logMAR = logarithm of the minimal angle of resolution; PVD = posterior vitreous detachment.

**Table 5:** Cytokines Associated with dry Maculae after the First Aflibercept Injection in Patients with Pachychoroid Neovasculopathy and Neovascular Age-related Macular Degeneration.

Cytokines	Pachychoroid neovasculopathy			Neovascular age-related macular degeneration		
	Dry (*n* = 10)	Wet (n = 8)	*p*-value*	Dry (n = 9)	Wet (n = 10)	*p*-value*
IL-1ra, pg/ml	124.2 (46.73–207.95)	78.7 (25.26–205.41)	0.72	62.05 (6.32–267.77)	173.00 (21.57–234.85)	0.69
IL-6, pg/ml	2.89 (1.97–7.80)	4.24 (2.26–19.32)	0.37	5.93 (2.04–46.56)	8.10 (4.51–11.30)	0.72
IL-8, pg/ml	5.53 (2.88–8.05)	4.90 (3.33–7.42)	0.92	6.46 (4.40–10.28)	6.41 (5.27–9.24)	0.96
MCP-1, pg/ml	178.69 (153.26–216.23)	216.68 (145.18–273.09)	0.33	253.35 (149.32–270.12)	205.81 (136.95–244.39)	0.07
MIP-1β, pg/ml	14.06 (10.60–22.84)	16.57 (15.08–18.33)	0.53	16.36 (14.45–31.49)	15.22 (13.34–20.19)	0.31
IP-10, pg/ml	152.98 (60.37–204.45)	155.31 (89.17–314.88)	0.33	170.46 (150.23–306.46)	201.03 (127.82–272.78)	0.83
bFGF, pg/ml	10.88 (4.51–9.34)	10.57 (9.34–20.02)	0.62	17.13 (1.72–24.11)	16.76 (11.94–23.80)	0.51
GM-CSF, pg/ml	46.19 (36.03–61.31)	47.03 (17.16–75.82)	0.86	56.61 (19.26–86.90)	56.61 (40.49–71.35)	0.83
VEGF-A, pg/ml	153.78 (103.81–175.77)	150.76 (99.53–245.43)	0.86	166.88 (147.07–201.60)	275.98 (207.66–298.00)	**0.02**
PlGF, pg/ml	4.30 (2.18–5.20)	4.02 (2.60–6.77)	0.56	3.67 (3.09–4.63)	5.99 (4.58–7.30)	**0.02**

Values are the median (interquartile range).

*P* < 0.05 was considered significant (bold).

*Wilcoxon signed-rank test.

IL-1ra = Interleukin 1 receptor antagonist; IL = interleukin; MCP-1 = monocyte chemoattractant protein 1; MIP-1β = macrophage inflammatory protein 1β; IP-10 = interferon-γ-induced protein 10; bFGF = basic fibroblast growth factor; GM-CSF = granulocyte macrophage colony-stimulating factor; VEGF-A = vascular endothelial growth factor A; PlGF = placental growth factor. Other cytokines were below the detection limit.

## Discussion

This is the first report to comprehensively analyse cytokine profiles in AH from PNV patients. By utilising multiplex bead immunoassays, cytokine profiles in AH for PNV patients were compared to those of nAMD patients, and the profiles were found to be useful, albeit only partially, for distinguishing the pathogeneses of these two diseases. AH is widely used to investigate retinal and choroidal diseases, such as nAMD^10,11,14^, diabetic retinopathy^15,16^ and retinal vein occlusion^17^. Performing anterior chamber paracentesis to obtain aliquots of AH is a safe and minimally invasive procedure^18^. Multiplex bead immunoassay makes it possible to simultaneously measure several cytokines in a limited volume, with excellent reproducibility. To examine whether PNV would possess distinct pathophysiological features from nAMD, the cytokines of AH in PNV, nAMD and control patients were comprehensively analysed. The following results were revealed: (1) VEGF-A in the PNV group was significantly lower than in the nAMD group but almost identical to that in the control group; (2) concurrent upregulation of proinflammatory cytokines was found in the nAMD group but was not significant in the PNV group; (3) the PNV, nAMD and control groups were distinguished by analysing the selected cytokines; and (4) there was a correlation between the features of the cytokine profiles and therapeutic response targeting VEGF-A and PlGF in the nAMD group, which was not the case in the PNV group. These results suggest that PNV and nAMD have independent pathophysiological features.

Since PNV is a new disease spectrum^1^, no definitive diagnostic criteria have been established to discriminate between PNV and nAMD. In this study, PC analysis showed that PNV can be distinguished from nAMD by four specific cytokines: MCP-1, bFGF, GM-CSF and VEGF-A. These results imply that the tentative diagnostic criteria adopted in this study were consistent with the pathophysiological features of PNV and nAMD based on cytokine profiles. The biological information from intraocular samples strengthened the optimal diagnostic criteria when using multimodal imaging.

From several reports on the clinical findings related to PNV^1,19^, it can be speculated that ischemia in the choriocapillaris or retinal pigment epithelium (RPE) dysfunction, resulting from compression of expanded choroidal vessels, may give rise to abnormal vascular networks. In contrast, there is only one report dealing with the molecular pathology of PNV^13^. Hata *et al*.^13^ found that the mean VEGF concentration in PNV was lower than that in nAMD, which agrees with the present findings, although their observed concentration of VEGF was relatively lower than that detected by the Bio-Plex assay used in our study. However, Hata *et al*.’s study was limited to the evaluation of VEGF, making it difficult to deduce the distinct roles of cytokines and angiogenic factors in nAMD and PNV. In nAMD, dysregulated inflammatory or angiogenic cellular stress may trigger the initiation of CNV development^20–22^. We recently reported on crosstalk between the RPE and macrophages in murine models, which was associated with the deterioration of chronic inflammation, resulting in the upregulation of VEGF, IL-6 and MCP-1 in association with the pathogenesis of early age-related macular degeneration (AMD)^23^. The VEGF family comprises seven members: VEGF-A, VEGF-B, VEGF-C, VEGF-D, VEGF-E, VEGF-F and PlGF^24^; its involvement, especially VEGF-A, in nAMD is known to be important for CNV development^25^. It is conceivable that other angiogenic factors may also be involved in the CNV pathology in PNV, in addition to VEGF.

It is known that inflammation plays a pivotal role in the aggravation of pathological progression of nAMD^26^. The Spearman’s rank correlation coefficient clearly indicated the presence of significant correlations among the proinflammatory cytokines IL-6, IL-8 and MCP-1 in the AH of the nAMD group, while the correlations among these proinflammatory cytokines in the PNV groups were significantly lower. The concentrations of proinflammatory cytokines, such as IL-6, IL-8, MCP-1 and IP-10, also tended to be lower in the PNV group compared to the nAMD group. Acute inflammation is the counter-response of a host to invasive detrimental cellular stress, whereas chronic inflammation is usually induced by tissue damage, leading to angiogenesis as a healing response^27^. Inflammation features are prominent in nAMD^28^, including pathological angiogenesis, and monocytes and macrophages play critical roles in this pathological angiogenesis^29^, producing various proangiogenic molecules, such as chemokines and cytokines, such as IL-6, IL-8, MCP-1 and IP-10^23^. This implies that pathological angiogenesis is more critical for the disease aggravation in nAMD than in PNV.

MCP-1 is involved in macrophage migration^30^ and has been reported to be associated with the formation and promotion of CNV in animal models^31^. Previous studies on the association of MCP-1 levels in AH with the pathogenesis of nAMD have described controversial results^11,32,33^. For example, Jonas *et al*.^33^ reported that higher MCP-1 was associated with classic or predominantly classic lesions in nAMD. In the present study, MCP-1 was significantly higher in the control group than in the PNV group, possibly due to only including type-1 CNV in the PNV and nAMD groups. Additionally, the control group AH yielded higher amounts of bFGF and GM-CSF compared to the PNV and nAMD groups, and bFGF was a potent angiogenic factor playing a pivotal role in the regeneration of various tissues^34^, while GM-CSF promotes the proliferation and differentiation of leukocytes^35^. Both are essential cytokines to maintaining physiological haematopoiesis. The microenvironmental changes in the anterior chamber due to pathological CNV formation in PNV and nAMD may account for the downregulation of these cytokines and the upregulation of VEGF-A.

In the present study, the relationship between cytokine profiles and the distinct therapeutic effects of anti-VEGF treatment on PNV and nAMD were examined. In the nAMD group, there was a significant negative association between the concentrations of VEGF-A and PlGF and the resolution of exudative change. In contrast, there was no significant association between cytokine and treatment response in the PNV group. In agreement with our findings, Funk *et al*.^36^ reported a correlation between increased VEGF in AH and persistent or recurrent macular oedema characterized by subretinal and/or intraretinal fluid in nAMD. Thus, the nAMD pathology may depend on the pathological angiogenesis displayed by VEGF-A and PlGF, while PNV may be less dependent on VEGF-A and PlGF, suggesting the possibility of the involvement of other angiogenic factors.

The present study has limitations that should be mentioned. First, the diagnostic criteria for PNV may not have been fully adequate because criteria for PNV have not been established, resulting in criteria being selected based on previous reports^1,7,19^. Second, cytokine profiles were only measured in AH. There are reports describing an association between nAMD and serum CRP upregulation^37^; thus, in future research, it may be necessary to compare data from AH to those from blood samples obtained simultaneously. Third, research to examine cytokine profiles in different stages of CNV with an increased number of cases is necessary to further rationalize the findings in this study.

In conclusion, this study is the first report to comprehensively analyse cytokines in the AH of PNV patients, a newly proposed disease concept. It was possible to differentiate PNV and nAMD by analysing specific cytokines in AH. The pathogeneses of PNV and nAMD may be different in terms of angiogenic factors, including VEGF-A and proinflammatory cytokines. The PNV and nAMD groups also exhibited disparate levels of dependence on VEGF-A and PlGF, showing distinct levels of responsiveness to anti-angiogenesis therapy. Further approaches seeking to elucidate the molecular features of angiogenesis or vasculogenesis in the pathology of PNV are needed to precisely measure multiple proangiogenic factors, in addition to VEGF, such as angiopoietin, bFGF, hepatocyte growth factor, IL-8, PDGF, MCP-1 and MIP-1β, which are produced mostly by macrophages.

## Methods

This was a prospective, comparative control study conducted in a single institution. The study was approved by the institutional review board of the Kyoto Prefectural University of Medicine, and the procedures were conducted in accordance with the tenets of the Declaration of Helsinki. Written informed consent was obtained from all participants after a detailed explanation of the study protocol, including AH collection.

### Study Population

All patients were recruited from the Department of Ophthalmology, Kyoto Prefectural University of Medicine University Hospital, between December 2015 and January 2017. The participants included 18 patients with treatment naïve PNV, 18 patients with treatment-naïve nAMD with type-1 CNV and 11 control patients without any retinal disease undergoing cataract surgery in both eyes. In the bilateral treated cases, left eyes were selected. The control patients were age and gender matched with the PNV and nAMD patients. Patients with a systemic history of diabetes, collagen diseases, cardiovascular disease, cerebrovascular disease, or systemic corticosteroid medications were excluded from the study. Eyes with a history of any other retinal diseases; uveitis; glaucoma, including ocular hypertension with any anti-glaucoma eye drops; any intraocular surgery, including cataract surgery; or high myopia (spherical equivalent < −6 dioptres or axial length > 26.5 mm) were also excluded from the study.

At the first examination, all patients underwent extensive ophthalmic assessment with refraction, BCVA testing with Landolt C charts, measurement of the axial length, slit-lamp biomicroscopy with or without contact lens, indirect ophthalmoscopy, colour fundus photography, fluorescein angiography (FA), indocyanine green angiography (ICGA), fundus autofluorescence (FAF) photography and spectral domain or swept source-optical coherence tomography (SD- or SS-OCT). OCT angiography was also performed to identify CNV if this was uncertain.

The clinical diagnoses of PNV or nAMD were based on fundus examination, fundus photography, angiography, SD or SS-OCT and OCT angiography by the researcher (N.T.). Axial length was measured using an interferometer (IOL Master, Carl Zeiss Meditec, Inc., La Jolla, CA), and colour fundus photography and FAF were obtained using a commercially available fundus camera system (TRC-50DX; Topcon, Tokyo, Japan) with a 50-degree field of view. FA and ICGA were performed with confocal scanning laser ophthalmoscope (Heidelberg Retina Angiograph 2; Heidelberg Engineering, Heidelberg, Germany). Cross-sectional images of the macular area were obtained using SD-OCT (RS-3000 Advanced System; NIDEK, Gamagori, Japan) or SS-OCT (DRI OCT Triton plus; Topcon, Tokyo, Japan). All patients underwent OCT angiography (DRI OCT Triton plus; Topcon, Tokyo, Japan).

**Figure 3:**
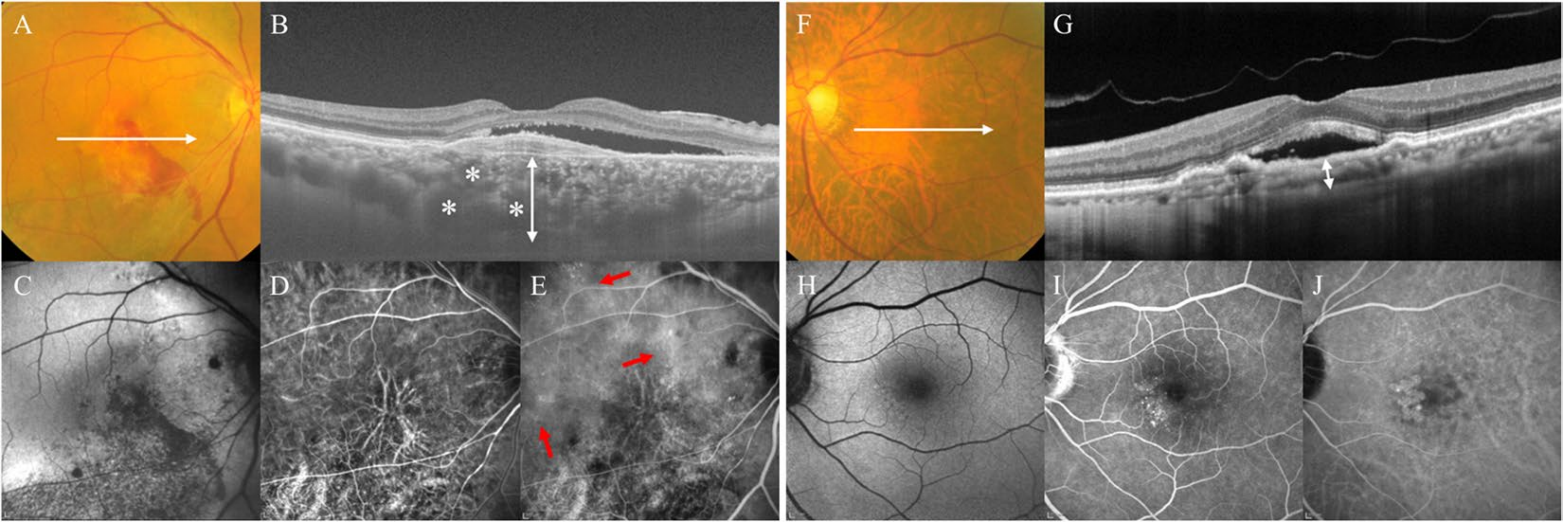
(**A**–**E**) Right eye of a 68-year-old male with pachychoroid neovasculopathy (PNV**)**. (**A**) Colour fundus photography revealed subretinal haemorrhage, serous retinal detachment (SRD) and absence of drusen. (**B**) Optical coherence tomography (OCT) along the white lines demonstrated type-1 choroidal neovascularization (CNV) with SRD. There is thickening of the choroid, with largely dilated choroidal vessels (asterisks) beneath the type-1 CNV. The subfoveal choroidal thickness was 654 µm (double-headed arrow). (**C**) Fundus autofluorescence (FAF) imaging showed a descending tract and granular hypoautofluorescent regions (arrows) in the maculae. (**D**) Indocyanine green angiography (ICGA) in the early phase revealed CNV and dilated choroidal vessels. (**E**) ICGA in the late phase revealed multifocal area of choroidal vascular hyperpermeability (CVH; arrows). (**F**–**J**) Left eye of a 77-year-old male with neovascular age-related macular degeneration (nAMD). (**F**) Colour fundus photography revealed SRD with soft drusen. (**G**) OCT along the white lines demonstrated type-1 CNV with SRD. The subfoveal choroidal thickness was 124 µm (double-headed arrow). (**H**) FAF imaging showed only the hypoautofluorescent region corresponding to a type-1 CNV lesion. (**I**) Fluorescein angiography revealed granular leakage of the dye in the maculae. (**J**) ICGA revealed well-defined plaque CNV in the late phase.

For the diagnosis of PNV (Fig. 3), the following criteria were used, based on previous reports^1,7,19,38^: (1) type-1 CNV detected in one or both eyes; (2) no or only nonextensive drusen (total area ≤ 125-µm circle) or hard drusen (≤63 µm) in both eyes (Age-related Eye Disease Study: category 1, no AMD)^39^; (3) CVH detected in the late phase of ICGA; (4) dilated choroidal vessels below type-1 CNV detected by ICGA and SD- or SS-OCT; and (5) the presence of CSC- or PPE-related RPE abnormalities independent of CNV lesions detected by FAF or a history of CSC. Neovascular AMD (Fig. 3) was diagnosed in the presence of type-1 CNV and other findings corresponding to AREDS levels 2, 3, and 4 (extensive hard drusen, soft drusen [intermediate, ≥63 and <125 μm; large, ≥125 μm], pseudodrusen, focal hyperpigmentation, or geographic atrophy)^39^, and elicited the following criteria: (1) no history of CSC; and (2) no characteristics of CSC or PPE.

The associations between the macular status and cytokines were statistically analysed after patients with PNV and nAMD were treated with intravitreal injections of 2 mg of aflibercept (Eylea; Bayer Healthcare Pharmaceuticals, Berlin, Germany). In these patients, the frequency of dry maculae was evaluated one month after the first aflibercept injection. A dry macula was defined as complete resolution of the intra- and subretinal fluid, whereas a wet macula was defined as the existence of any intra- or subretinal fluid detected by vertical and horizontal SD- or SS-OCT scans.

### Collection of Aqueous Humour

AH was obtained at the beginning of surgery in control patients and before intravitreal injection of aflibercept in the PNV and nAMD patients. Before paracentesis of the anterior chamber, the eyes were sterilised with povidone iodine and washed with saline. A specially designed 30-gauge needle integrated with a disposable pipette (Nipro, Osaka, Japan), as described in a previous report, was used to withdraw AH^18^. Approximately 150 µl of AH was collected and immediately stored at −80 °C until cytokine analysis.

### Multiplex Analysis of Cytokines in Aqueous Humour

The concentrations of 30 cytokines in AH were analysed using a Bio-Plex multiple assay (Bio-Plex Multiplex Immunoassay System; Bio-Rad, Hercules, CA). Measured cytokines were bFGF, exotoxin, granulocyte colony-stimulating factor, GM-CSF, interferon-γ, IL-1β, IL-1ra, IL-2, IL-4, IL-5, IL-6, IL-7, IL-8, IL-9, IL-10, IL-12, IL-13, IL-15, IL-17, IP-10, MCP-1, MIP-1α, MIP-1β, platelet-derived growth factor BB; regulated upon activation normal T-cell expressed and secreted tumour necrosis factor-α, VEGF-A, VEGF-C, VEGF-D and PlGF. The analysis was performed according to the manufacturer’s instructions. Standard curves were generated using the Bio-Plex TM 200 System (software version 6.1; Bio-Rad Laboratories).

### Image Analysis

Colour fundus photographs were used to grade large drusen according to the simplified severity scale for AMD from the Age-related Eye Disease Study^40^. Subfoveal choroidal thickness was calculated by measuring the distance between the hyperreflective line corresponding to the Bruch membrane beneath the RPE and the inner surface of the sclera with the calliper function of the SD- or SS-OCT. The presence of complete PVD was determined using the SD- or SS-OCT images. Eyes were defined as having complete PVD if detached vitreous was identified in vertical and horizontal scans or if there was a total absence of the posterior hyaloid. The diagnosis of polypoidal lesions was confirmed by the presence of dilated polyps at the end of the branching vascular network on ICGA^41^. The presence of CVH was determined by two retinal specialists (i.e., N.T. and T.Y.) and appeared as multifocal hyperfluorescence in the middle and late phases of ICGA. The two specialists were blinded to the clinical findings, and CVH was determined only when both retinal specialists met the same judgment. The GLD was measured manually using a ‘measure lesion’ tool in the software embedded in Heidelberg Retinal Angiograph 2 as the lesion dimension covering the area of possible neovascular membrane, including the areas of dye leakage, pigment epithelial detachment and subretinal haemorrhage on FA and colour fundus photography. CNV lesion size was calculated manually in each early ICGA image using the ‘draw lesion’ tool of the software embedded in Heidelberg Retinal Angiograph 2.

### Statistical analysis

Statistical analysis was performed using the JMP Pro V.11.2.0 software (SAS Institute, Cary, North Carolina, USA) and R statistical package (version 3.4.1 for Windows, Vienna, Austria) programmes. A *p-*value less than 0.05 was accepted as statistically significant. Unless otherwise stated, the results were expressed within median and interquartile ranges, and categorical data were assessed using Fisher’s exact test. The Kruskal-Wallis test was employed to compare multiple groups, and the Wilcoxon signed rank test was used to compare the means of the quantitative variables between two independent groups. The statistical significance of the differences in cytokine concentrations among the three groups was calculated using the Kruskal-Wallis test, before cytokines with significant differences were analysed using the Steel-Dwass method. Spearman’s rank correlation coefficients were used to assess the relationships between cytokine concentrations in the PNV and nAMD groups, while PC analysis was used to compare the cytokine characteristics of each disease and to clarify differences between them.
